# Crisis Management Tasks in Dutch Nursing Homes During the COVID-19 Pandemic: A Longitudinal Interview Study

**DOI:** 10.1177/10775587221150477

**Published:** 2023-02-01

**Authors:** Jeroen van Wijngaarden, Marleen de Mul, Kees Ahaus

**Affiliations:** 1 Erasmus University Rotterdam, The Netherlands

**Keywords:** crisis management, COVID-19 pandemic, resilience, nursing homes

## Abstract

The COVID-19 pandemic hit long-term care, and particularly nursing homes hard. We aimed to explore how crisis management goals and tasks evolve during such a prolonged crisis, using the crisis management tasks as identified by Boin and ‘t Hart as a starting point. This longitudinal, qualitative study comprises 47 interviews with seven Dutch nursing home directors and a focus group. We identified two phases to the crisis response: an acute phase with a linear, rational perspective of saving lives and compliancy to centralized decision-making and an adaptive phase characterized by more decentralized decision-making, reflection, and competing values and perspectives. This study confirms the usability of Boin and ‘t Hart’s typology of crisis management tasks and shows that these tasks “changed color” in the second phase. We also revealed three types of additional work in managing such a crisis: resilience work, emotion work, and normative work.

## Introduction

When the COVID-19 pandemic started in 2020, hospital care stood center stage. The main focus was how hospitals could create enough ICU capacity to treat severely affected individuals. Scarce resources such as personal protective equipment were prioritized for hospital use. At the same time, nursing homes were also affected. After the first COVID-19 wave passed in May 2020, the Dutch media reported a “silent disaster” in nursing homes in the Netherlands. At that time, 46% of all COVID-19-related deaths had occurred in a nursing home (Rijks Instituut voor Volksgezondheid en Milieu [RIVM], 2020). This was also reported in many other countries in the first months of the pandemic (Dykgraaf et al., 2021). Nursing homes are particularly susceptible because they primarily house the most vulnerable people, often in the last stage of their lives. In face of the pandemic, nursing home directors became crisis managers overnight, dealing with uncertainty and scarce resources, and balancing the needs of their residents (and families) with those of their staff. In this article, we study the challenges faced by nursing home directors during the first year of the COVID-19 pandemic and the tasks they needed to perform to manage the crisis.

Pandemics, such as COVID-19, have been characterized as low-chance, high-impact crises or long-shadow crises (Boin & ‘t Hart, 2010; Lloyd-Smith, 2020). They create a high level of uncertainty and urgency, and have an exceptional magnitude and prolonged impact (Boin & ‘t Hart, 2003; Nohrstedt et al., 2018; Steen & Morsut, 2020). Even after the first outbreak struck Wuhan in 2019, most European countries were still surprised at the impact the virus had. Tourists brought the virus to the Netherlands after visiting Italian ski-resorts in January and February 2020. Between March and April, infection rates spiked, and intensive care units (ICUs) were at risk of becoming overwhelmed. A countrywide lockdown helped to temper the first wave and ICUs were able to manage the infection rates from May to October 2020, until the next wave hit.

The COVID-19 pandemic impacted the way organizations and their leaders manage such a crisis, as they had to continuously “prepare for and respond to unscheduled, undesirable, urgent and threatening contingencies” (Boin & ‘t Hart, 2010, p. 357). In the literature, crisis management has three main goals: save lives, protect (critical) infrastructure, and restore trust in (public) institutions (Boin et al., 2013). To achieve these goals under uncertain conditions, Boin and ‘t Hart (2010) have identified crisis management tasks that need to be performed, based on so called single-event crises. They did an analysis of “the most robust” existing studies on crisis events to identify “best practices” in crisis management (Boin & ‘t Hart, 2010, p. 358). They made a distinction between two levels on which crisis management tasks need to be performed, namely, strategic and tactical/operational levels. Strategic crisis management involves critical decision-making at the highest level, providing guidance to the general public and the response network. This incorporates tasks like ‘meaning making”—which involves “providing persuasive public accounts of what is happening and why”—and “account giving”—which involves “managing the process of expert, media, legislative and judicial inquiry and debate” (Boin & ‘t Hart, 2010, p. 359). These tasks were primarily the responsibility of national governments during the COVID-19 pandemic, especially during the first wave. Tactical/operational crisis management involves a number of tasks or response challenges focused on minimizing the consequences of the crisis and providing relief to those affected (Boin & ‘t Hart, 2010). Nursing home directors played an important role at this level. The tasks of tactical/operational crisis management are presented in Table 1, based on Boin and ‘t Hart (2010) and incorporate, among others, using available resources to *contain and mitigate* the threat. However, the COVID-19 pandemic is not a single-event crisis but a crisis with a massive and prolonged impact to which the traditional phases of precrisis (prepare), crisis (respond), and postcrisis (recover) (Steen & Morsut, 2020) do not seem to apply, because the start and end are not clear, and the intensity of the crisis comes in waves. Therefore, we expected that Boin and ‘t Hart’s typology of tactical and operational tasks of crisis management might need further elaboration in this new context. Specifically, we hypothesized that goals and tasks would evolve during the crisis.

**Table 1.** Crisis Management Tasks on the Tactical and Operational Level (Boin & ‘t Hart, 2010).

Task	Description
Diagnosing and deciding	Forming an accurate picture of the nature and extent of the threat and/or damage under conditions of time pressure and incomplete information; choosing a sensible and feasible initial response approach; and continuously updating both in light of changing circumstances or additional information becoming available
Mobilizing and organizing	Soliciting the types and levels of operational resources necessary to meet the demands of the situation in a speedy yet orderly fashion, and deploying them in a timely and orderly fashion
Containing and mitigating	Using available resources effectively and efficiently to contain (and if possible, reduce/eradicate) the agent(s) of threat and destruction so as to minimize the damage to lives and property
Informing and empowering	Transmitting accurate, timely, and actionable information upward, outward, and downward within the crisis response structure, as well as to relevant citizens and communities, designed to enable these actors to make informed crisis response decisions within their respective domains of involvement
Coordinating and collaborating	Making sure different units, organizations, and disciplines involved in frontline crisis responses work together effectively and in a sustainable way, both within and across the public and private/community sectors

In this article, we present the results of a qualitative, longitudinal study on crisis management in nursing homes in which we followed crisis managers over several months during the COVID-19 pandemic. Our first aim is to conceptualize crisis management in nursing homes and to verify if the crisis management tasks identified by Boin and ‘t Hart (2010) are also relevant for this type of crisis in nursing homes. Second, we aim to explore how crisis management goals and tasks evolve during a prolonged crisis with high impact. To achieve these aims, we formulated the following research questions:

**Research Question 1 (RQ1):** Which tasks did nursing home directors need to perform because of the COVID-19 crisis?
**Research Question 2 (RQ2):** Which goals did they pursue?
**Research Question 3 (RQ3):** How did these tasks and goals change during the crisis?

## New Contributions

Existing studies on crisis management are primarily based on the analyses of single devastating events at a particular moment in time, much less on crises with a prolonged impact like COVID-19. That is why little research has been done on how crisis management goals and tasks evolve during such prolonged crises (Boin et al., 2013). At the same time, crisis management in nursing homes is hardly studied. Using the goals and tasks identified by Boin and ‘t Hart (2010) for crisis management as a reference point, we will show how these develop and “change color” during the different phases of the crisis. Furthermore, we will show the importance of different types of work, namely, resilience work, emotion work, and normative work for performing these tasks in nursing homes in this type of crisis.

## Methods

### Context/Setting

This research was conducted in nursing homes in the Netherlands. These homes typically care for older adults in the final phase of life: the average stay is 9 or 10 months, until their death. Twenty percent of nursing home residents stay less than 3 months and 50% stay longer than 18 months. The inhabitants of Dutch nursing homes are usually frail and have multiple morbidities and a large group suffers from dementia, which lowers their quality of life (Hartog et al., 2016). Nursing homes are often part of larger, regional elderly care or long-term care groups, with several locations in a region, or even several sites in one town. Many of these groups also deliver home care services. There are about 650 organizations in the Netherlands that provide nursing home care, at around 1150 locations, with approximately 130.000 beds in total (Inspectie voor de Gezondheidszorg, 2020; TNO, 2019). Nursing homes are financed through the Long-Term Care Act, which covers stay in the facility, personal care, nursing care, and day programs or day treatment (Ministry of Public Health, Welfare and Sport, 2016). For curative care, the Netherlands has a system of competing health insurers. The long-term care uses a single payer system, for which the country is divided in eight regions. The regionally dominant curative care insurer takes on the role of health care purchaser for long-term care, including nursing home care. Nursing home staff consists of geriatricians, therapists, registered nurses, and a large group of nursing attendants. Nursing homes employed in 2019 approximately 1672 geriatricians, making each responsible for the care of around 80 patients (Stichting Capaciteitsorgaan voor Medische en Tandheelkundige Vervolgopleidingen, 2019). Nursing homes are traditionally led by general managers. Each organization has a board of directors with location or team managers managing the different nursing home sites. In contrast with hospitals, the “medical model” is not dominant, and clients are seen as residents who “also” have medical needs (White-Chu et al., 2009). Geriatricians are therefore mostly not involved in management but do have an important advisory role. Nursing homes also have an advisory board in which the nursing staff is represented.

### Design

The design is a longitudinal qualitative study consisting of interviews with seven nursing home directors (most of whom are board members of the nursing homes) and a focus group.

A total of 47 interviews were conducted between March and July 2020, with each respondent undergoing an average of 6 interviews (minimum 2 interviews, maximum 11 interviews). Interviews were structured according to a “dynamic” topic list, which was tailored to the then current pandemic situation. During the interviews, data were collected on the current affairs in the nursing homes (what the directors had been doing in the past few weeks) and directors reflected on their role and the dilemmas they faced.

All interviews were conducted by phone at a time convenient for the respondent (usually after office hours) and were audio-recorded and transcribed. Interviews lasted between 26 and 67 min (average 40 min). In total we collected over 32 hr of interview data.

The interviews stopped after the first wave. To understand how the work of the crisis managers had changed between the first and second wave, and how the second wave was perceived, a 2-hr focus group was organized in February 2021, which was attended by five of the original respondents. One respondent was not available and was represented by a colleague. This focus group also member checked the first analysis.

As a preparation to the focus group, we sent a summary of our preliminary findings to the participants and asked them to comment on this analysis—we asked if they recognized the issues from their own organization and which issues they had faced during the second wave. During the focus group, we discussed the traits of the second wave and current preparations for the third wave that was expected. The responses were compared between organizations and regions. The respondents also reflected on the dynamics and dilemmas of central steering by the government and regional collaboration. The focus group session was conducted online because of COVID-19 restrictions, and was video-recorded and transcribed. All respondents gave written consent to participate in the interviews and focus group.

### Respondents and Data Collection

Participants were experienced leaders, recruited from our own network and via snowballing. The participating nursing homes were spread across the country to assure the cases were representative. This was important because the pandemic varied in severity across the Netherlands; crisis management in the north may have different challenges and tasks to those working in the south. The pandemic also had different impacts on different nursing homes—some had many cases while others had none. Table 2 presents the respondents’ characteristics.

**Table 2.** Respondents, Interviews, and Context.

Respondent	Gender	Role	Region	Size of nursing home care organization	Number of interviews	Focus group attendance
R1	F	Board member	South-West	32 locations for somatic and dementia care, 1,000 clients	4	Y
R2	F	Board member	South-West	Five nursing homes, 500 clients	2	N
R3	F	Crisis manager	North	Eight nursing homes, nine small-scale dementia homes, 1,200 clients	11	Colleague (R8)
R4	F	Board member	South	14 nursing homes, 1,300 clients	4	Y
R5	M	Board member	North-West	Six nursing homes, one small-scale dementia home; 500 clients	5	Y
R6	M	Board member	North-East	Six nursing homes, four small-scale dementia homes; 700 clients	12	N
R7	M	Board member	North-West	One nursing home; 100 clients	9	Y

### Data Analysis

Transcripts from the interviews and focus group and written feedback from the member check were analyzed following an abductive approach (Tavory & Timmermans, 2014). One author (K.A.) performed the first iteration using Boin and ‘t Hart’s (2010) description of the tasks and goals and the concept of resilience to deductively code the data using Atlas.ti. All authors performed the second iteration, which was refined task-by-task by adding open codes on the COVID-19-specific content and timing of the quotes. Tasks were divided among the three authors, which sometimes changed the initial coding of the task. This was followed by axial coding, where codes were clustered thematically, chronologically, and by type of work performed by the crisis manager. For each task, two authors independently performed the axial coding using Microsoft Excel and the analysis was discussed until consensus was reached. This analysis revealed types of work that were not mentioned explicitly by Boin and “t Hart (2013) and showed that tasks evolute (‘get a different color”) over time because goals change between—what we distinguish as—the acute phase and the adaptive phase of the crisis response.

## Results

When COVID-19 arrived in the Netherlands, nursing homes were not prepared for the COVID-19 pandemic. Although high infection rates had already been reported in the south of Europe (especially in Italy), nursing home directors did not expect the virus to arrive in the Netherlands as quickly as it did. The virus was introduced to southern parts of the Netherlands by tourists returning from skiing holidays in Italy. Then, during the traditional carnival festivities in the beginning of February, the virus spread fast, mainly in the southern parts of the Netherlands. Although the north of the Netherlands was not hit by this first wave, all our respondents felt they needed to respond fast to this crisis, with little knowledge and information, to prevent further infections and save lives:

I was really worried for a while, because I thought our management team was not equipped to deal with this. At the time they were all over the place, everybody was micromanaging. And I thought, oh no, if it is going to be like this, while this crisis just started, it is not going to work. That was really a personal wake-up call. (R1, March 2020)

According to our respondents, this first phase of the COVID-19 crisis response was characterized by a number of traits. The first trait was a strong focus on the goal of preventing further spread of the virus to *save lives*. The government decided to close nursing homes to outside visitors and volunteers. Often, inhabitants could no longer go outside, except in closed gardens. Most nonessential functions and activities were stopped, such as daytime activities and trips to the indoor restaurant and the hairdressers:

It was on a Thursday evening at 7pm (when the government decided to close all nursing homes) and then everybody needed to leave immediately. Those people (visitors) had not watched the news and had no idea what was happening, but they were sent home. This was something our staff members should have handled with more care. They could have said, please drink one cup of coffee with your mother or father before you leave because the government said you’re not allowed to see him or her for a while. (R3, March 2020)

The second trait was *solidarity* between all stakeholders in long-term care, including health care purchasers. In most regions, structures for cooperation already existed, but these were mostly weak because nursing homes are to some extent in competition with each other. Now in a short time, the stakeholders found each other and started to develop policies and procedures, for example, to procure and share scarce resources such as personal protective equipment. Health care purchasers basically told the nursing homes to do what they could to protect lives and that all costs would be reimbursed:

I think that, in such exceptional circumstances, everybody knows they’re not going to make it alone, and that they need each other. And also the willingness, and I feel that everywhere, to look beyond the interests of your own organization and focus on the collective interests. (R3, April 2020)

The first thing that they (healthcare purchasers) did and did well, was reassuring the institutes (nursing homes) that the crisis would have no negative financial effects (they would be compensated), which was very nice. (R7, April 2020)

The third trait was *compliancy* with centralized decision-making. The Netherlands is usually characterized by decentralized structures, with low power distance and high individualism (Hofstede, 1984). However, in this first phase, our respondents, their employees, and their residents and families were mostly willing to comply with (and were even expecting) more centralized decision-making on the national level. Furthermore, the advice given by the National Institute for Public Health and the Environment (RIVM), the primary advisory board on COVID-19 in the Netherlands, was mostly accepted and followed:

I think we still need to keep central control. We were very strong on central control, maybe a bit too strong because of our fear of scarcity of ICU beds. I think everybody was scared. But to go back now to decentralized control with what I think might be the outcome, is too soon for me. (R4, May 2020)

Finally, there was a strong focus on *action*. Our respondents reported having little time for reflection and the focus was on ‘*act, act, act’*: building decision-making structures, developing procedures and protocols, procuring extra resources, creating COVID-19 units, and reserving extra beds for COVID-19 patients:

It was about urgency; things needed to be solved quickly. Many people needed to make good decisions, without really having the time to think about it. That also meant no time for debate. (R6, April 2020)

When the first wave flattened out in May 2020, the pandemic response seemed to enter a new phase. Although the virus was not under control and more waves were imminent, the crisis response changed according to our respondents. The second wave did not hit the Netherlands until October 2020, which gave people the summer of 2020 to reflect on the first wave and prepare for the next.

And of course, we are now preparing an evaluation, today the board of directors has agreed to this. So yes, we are going to think about what we have learned and also what good things we need to preserve. (R3, May 2020)

Most nursing homes now had their structures, protocols, and procedures in place and there were no longer shortages in personal protective equipment. The Dutch government decentralized decision-making, allowing the nursing homes more freedom in choosing when and how to open, close, and protect their facilities. At the same time, compliancy decreased strongly. Directors, employees, and families started to question past and current decisions and advice. Issues and goals other than “saving lives” were brought to the fore, including the wellbeing of patients and staff who had suffered emotionally and physically from the pressure. Many questioned whether closing down the nursing homes was a wise decision. Residents of nursing homes in the Netherlands are mostly very frail and in the final stage of their lives, and many thought that the priority should be ensuring a good quality of life rather than saving lives:

The strict visitors’ rules are on the one hand necessary to prevent infections . . . But it is a devil’s dilemma because quality of life is very important for us. (R2, June 2020)

Most people that are cared for in nursing homes have a life expectancy of nine months on average . . . That begs the question, when your life expectancy is only nine months well then . . ? Well, you may die sooner (because of COVID-19), shit happens, but with your loved-ones you would at least have quality of life and now without them you do not. (R3, April 2020)

The decisions made by the Dutch government and RIVM were increasingly debated and questioned in Dutch society. After *decentralization*, these *multiple values* and *multiple perceptions* increased the pressure on nursing home directors to make well-informed decisions and to justify these decisions:

We really try to properly explain why we chose each of our policies or why we deviated (from existing policies). (R3, May 2020)

This increased responsibility on individual directors, put pressure on solidarity between directors and on the relationship between directors and geriatricians. Although many nursing home directors still came together to align their policies, some started making choices that were good for their nursing homes but not so good for others, such as being much more lenient toward allowing visitors than others.

While directors now needed to make their own decisions often under time pressure, geriatricians expected to be involved again:

I needed to make speedy decisions (to unlock the nursing home). So, i said we need to allow the hairdresser to return, we need to decide this and that . . . go-go-go. But I guess we were not diligent enough. We did not have the right conversation and the geriatricians really bucked (like a bronco). They were really furious, and we had a big riot. (R3, May 2020)

At the same time, new waves posed new challenges, such as the shortage of personnel because many were sick, followed by the debate on how and who to vaccinate. Nursing home directors were consistently *adapting* their policies and protocols to these challenges and there was a constant shift between decentralized (between waves) and centralized (during waves) decision-making. Finally, the prolonged nature of the COVID-19 pandemic also negatively affected the emotional and physical wellbeing of employees and residents:

This second wave runs a more capricious course especially for our employees. I think this is characteristic of the second wave. Employees get sick and get sick for a long period of time. (focus group, February 2021)

During the interviews, respondents were asked how they dealt with the pandemic and how these responses changed over time and why. We noticed that the tasks undertaken in the first phase of the crisis response could be categorized as described by Boin and ‘t Hart (2010) and that the work could be covered by the definitions given for these tasks. However, we noticed a clear shift in the tasks between the two response phases—the tasks ‘changed color” according to the different traits of these phases. Table 3 shows these “changes of color” for each task with concrete examples from our respondents.

**Table 3.** How Crisis Management Tasks Change in Color.

Task	Related work in the acute phase	Related work in the adaptive phase
Diagnosing and deciding	• Close monitoring of infections and availability of PPE. • Strictly following governmental decisions and adjusting protocols according to national guidelines. • Decisive action in scaling up and down activities and services (e.g., testing, COVID-19-unit, COVID-19 bed capacity, and daytime and indoor activities).	• Dealing with new uncertainties (e.g., setting up visits and vaccinations, dealing with new virus variants), which require adaptation of protocols and procedures. • Reflexive action, trying to see what actions best fit local circumstances and expectations taking wellbeing of clients and personnel into account. • Setting up ethical committees.
Mobilizing and organizing	• Solving acute shortages of PPE using national structures and own networks. • Much solidarity among personnel and regional partners; putting in extra time and effort to deal with the crisis. • Development of regional cooperation to obtain and share resources. • Installing a crisis organization: Setting up central Corona teams with the involvement of doctors and nurses.	• Dealing with shortage of personnel because of fatigue and illness; some deploy army personnel. • Organizing tests and later on vaccinations. • No shortage of PPE anymore. • Crisis organization is in place and decentralized.
Containing and mitigating	• Containing the virus and tracing close recent contacts of anyone who tests positive. • Drawing up tailored contingency plans for different scenarios. • Managing concerns of staff being insufficiently protected and feelings of guilt about infecting residents.	• Balancing containment of the virus and wellbeing of residents. • Following protocols and procedures to minimize threats. • Scaling up and down activities depending on threat.

*Note*. PPE = personal protective equipment.

We identified three types of work, related to multiple tasks, that are relevant to the COVID-19 crisis but were not explicitly mentioned by Boin and ‘t Hart (2010). These are resilience work, emotion work, and normative work.

*Resilience work* increases the ability of the organization to adapt to changing challenges during the crisis. This allows the organization to better anticipate and respond to challenges and to readjust as new waves come and go. During the first wave, nursing home directors in the south of the Netherlands were primarily busy dealing with infected patients and preventing the virus from spreading further. But still they were also thinking about protocols that could help them with new infections. In the north of the Netherlands, nursing home directors had more time for resilience work during the first wave as there were considerably fewer infections:

We are now through the first phase, we have prepared for many different scenarios, and it also makes a difference that it is now part of our regular business operations. We are able to adapt now, we can also restart normal activities. (R6, May 2020)

*Emotion work* manages the emotions of individuals working in the nursing home. Nursing home directors gave many examples of emotion work. They described their own fears and insecurities as well as those of employees, clients, and families and how they dealt with these feelings during the first phase. These emotions were managed by giving information, being visible, and acting decisively. The first lockdown had a major impact on all individuals in the nursing home:

Employees have witnessed distressing situations of increasing loneliness of elderly residents who did not understand the situation. Continuously needing to explain . . . your son is not allowed to visit, I understand it is really difficult, but it is not possible right now, which is of course difficult . . . People start working in care because they want to help clients and now they needed to act like policy officers, forbidding all kinds of things; this is really new for our employees. (R6, May 2020)

Nursing home directors started organizing social support, such as a social service that monitors wellbeing of personnel, or a support line people can call. They also held meetings to show support and create trust:

We have a support line, which you can always call. It is available seven days a week during the day, operated by our medical psychologists. (R3, May 2020)

I think the different tasks you identified are nice but; c’est le ton qui fait la musique (it is the tone that makes the music); the personal style of a director is important and makes all the difference. (focus group, February 2021)

Remorse was also a common emotion. Employees felt guilty for bringing the virus into the nursing homes while they had to stay closed to visitors. Nursing home directors felt guilty when they made decisions that had a negative impact on the residents and staff. Nursing home directors told how they shared these experiences with other directors to deal with their emotions:

It is also true that current contaminations are caused by healthcare workers, because clients are no longer allowed to have contact with their families. So basically, the only contact they have with the outside world is through healthcare staff. Several nurses and carers were not pleased when this was mentioned. They said: “we do our absolute best, we run risks and now we will soon also be blamed for people dying.” (R6, April 2020)

Managing emotions became more complicated as compliance decreased and different values and perspectives surfaced. Nursing home directors had to deal with indignation and anger not only from clients and family but also from the media and the general public:

The past couple of weeks were more difficult than before. We had to deal with many angry people and needed to explain each time how it is a balancing act between allowing freedom and limiting risks. (R5, June 2020)

*Normative work* organizes moral reflections and judges value to guide decision-making. Normative work was especially relevant in the second phase of the crisis. In the first phase, decision-making was centralized and focused on saving lives and the government and the RIVM therefore played a dominant role in making value judgments. However, during the second phase nursing homes could make their own choices and needed to deal with multiple perspectives and values. Most nursing home directors organized ethical meetings and set up ethical committees (involving among others geriatricians and nurses) to deal with this new challenge:

You have to consider both the health of our clients and let’s say the fun they have in life. That is an ethical debate and that is why we set up an ethical committee. At the same time, we need to be realistic, some things are not allowed based on the emergency ordinance. That makes our life a bit easier now, as we can also hide behind this emergency ordinance. But there will be more room to maneuver in the future, which we will need to give substance to. (R6, May 2020)

Regarding clients with dementia, it is difficult to tell them not to interact with others. It is safer to move and isolate them. But moving someone with dementia is really not good for the client. So again, an ethical dilemma, now between the interests of an individual and those of the group. (R1, May 2020)

## Discussion

The COVID-19 pandemic is different from most crises studied in the literature on crisis management because it does not involve a single devastating event at a particular moment in time, but rather comes in waves over an extended period (Bundy et al., 2017). Therefore, it does not follow the simple order of precrisis, crisis, and postcrisis that has often been described in the literature on crisis management but instead goes through different phases (Steen & Morsut, 2020). Traditional models on crisis management do not fully apply to this type of crisis. These models have a strong linear, rational, and hierarchical perspective, with the view to “identify and fix the problems in inputs and operations that lead to ineffective outputs” (Bundy et al., 2017; Kahn et al., 2013; Williams et al., 2017). This approach to crisis management can also be recognized in medical journals (Abdi et al., 2022; Geerts et al., 2021; Pring et al., 2021). However, according to our study, this perspective was only relevant during the first phase of the COVID-19 pandemic response. During this first response phase, the focus was primarily on the goal of saving lives and there was much *compliancy* with centralized decision-making and a strong emphasis on action to solve problems such as shortage of personal protection equipment. Nobody had experience planning for a pandemic (Usher et al., 2021). However, when the pandemic response entered the next phase, this traditional perspective no longer applied. Decision-making became less hierarchical and linear and more decentralized and iterative in nature. Instead of focusing on saving lives, other issues and goals became important, such as re-establishing the wellbeing of clients and employees. Different stakeholders (employees, clients, families, media) started to question previous decisions and new waves brought new challenges. Instead of a precrisis, crisis, postcrisis order, the adaptive phase basically consisted of consecutive episodes of precrisis (preparing for the next crisis) and crisis. These episodes could vary between regions and even between nursing homes, as infection rates varied. As a result, crisis management in nursing homes needed to become much more responsive and adaptive.

To help identify and conceptualize the tasks nursing home crisis managers and board members needed to perform during the COVID-19 crisis, we used the typology of Boin and ‘t Hart (2010) as a starting point. They identified, based on existing literature, a number of key tasks that need to be performed during a crisis on tactical/operational level, namely, diagnosing and deciding, mobilizing and organizing, containing and mitigating, informing and empowering, and coordinating and collaborating. We found that this typology could incorporate all the tasks mentioned by our respondents. However, the tasks were described by Boin and ‘t Hart (2010) for a single-event crisis. We found that in a prolonged or long-shadow crisis, like the COVID-19 pandemic, these tasks ’change color’ in the second “adaptive” phase and need to be described differently to reflect the necessity to learn and adapt and deal with multiple values and perspectives. This also related to the fact that the Dutch central government partly decentralized decision-making after the first phase, giving some responsibility—and hence also accountability—back to the nursing homes.

We identified three types of work related to multiple tasks that were relevant to the COVID-19 crisis. These were resilience work, emotion work, and normative work. Resilience work has been discussed previously by Boin and ‘t Hart (2013) and in other publications on crisis management, but mostly in pre- or postcrisis and in a separate task (Williams et al., 2017). In our study, we found that resilience work was an integral part of each task during the COVID-19 crisis and an ongoing process. Resilience work helped the organization adapt to the changing challenges and to anticipate, respond, and readjust as new waves came and went. A recent study from Norway showed how nursing homes invested in different innovative solutions during the first phase of the COVID-19 crisis to enhance resilience (Lyng et al., 2021). Emotion and normative work were especially important in the second phase of the crisis response. During the first phase, the need to save lives dominated and decisive action and clear communication were important strategies for managing fear.

Other studies have shown that communication between management is important for reducing negative emotions during a crisis (Guzzo et al., 2021). Things changed in the second phase of the crisis response when different values came into play and people started to wonder if and when the crisis would end, creating a mixture of emotions. During this time, nursing home directors had to put more time and energy into managing the emotions of others and themselves using multiple strategies, and in organizing moral reflections and making value judgments to guide decision-making. Other authors have also highlighted the importance of reflexivity when making decisions during a crisis (Schippers & Rus, 2021). Several authors have described the dilemmas and emotions of family members who were isolated from their loved-ones living in nursing homes during the COVID-19 pandemic (Madrigal et al., 2021; Nash et al., 2021). These findings suggest that emotion work and normative work are an important part of crisis management (Bundy et al., 2017). This relates also to discussions in the crisis management literature on learning (Bundy et al., 2017). During the acute phase learning focused partly on developing, but mostly on implementing interventions to deal with infections and prevent the (further) spread of infections. In this phase vicarious learning was organized; learning from other nursing homes and from experiences in hospitals. However, there was little time and opportunity (given also the centralized governmental decision-making) to properly reflect on the effects of the measures taken, for example, on the proper use of personal protection equipment. At the same time, as Haunschild et al. (2015) also noticed during other types of crises, learning had a quite narrow focus (especially on safety) at the expense of other values. This however changed in the nursing homes during the acute phase, when more moments for reflection were organized, involving more people with different perspectives, related to multiple values.

Our findings also suggest that leadership styles of the nursing home directors changed during different phases and episodes of the pandemic. During the acute phase, decision-making seems quite (consultive) autocratic in nature, with more focus on managing tasks that need to be performed (instead of relationships). This is in line with what many authors say about leadership in times of crisis (Bundy et al., 2017). During the acute phase professionals lost much of their autonomy. Our directors advised by a small group of decision-makers (location managers and often geriatricians) primarily decided how to proceed. Directors say that this was required because of time pressure and the high stakes involved. Some suggest it also served an emotional need for decisive leadership. At the same time, it was strongly influenced by the fact that the government made some of the key decisions regarding the use of PPE and the lock-down. The directors were merely expected to copy and implement these decisions. This changed during the adaptive phase, when crisis decision-making decentralized. Although, professionals still had to adhere to many protocols and rules, more stakeholders were involved including client representatives, for example, in the ethical boards. The role of the directors became much more focused on finding consensus and on managing relationships (instead of tasks) through empowerment and emotion work. However, during crisis episodes, when new pressing challenges arose, for example shortage of staff, directors had to take up a more directive role to specifically deal with those challenges. In general, our respondents suggest that their leadership style was contingent to the circumstances, taking into account urgency, risk, emotional needs, normative pressures and the role of the government (Belrhiti et al., 2018).

Our study has highlighted specific lessons for the health care sector. On one hand, the health care sector has dealt with the COVID-19 pandemic with flexibility and solidarity—health care purchasers and health care providers who were normally competitors found a common ground and worked together. On the other hand, hospitals dominated the health care sector and demanded the most attention. Policy makers forgot about nursing homes during the first wave of the pandemic and could have made better use of the capacity and knowledge of nursing home staff (Sunner et al., 2021; Usher et al., 2021). The goal to save lives, while dominant in a hospital, is not the dominant value in nursing homes, where most residents are nearing the end of life and want to enjoy their final days as much as possible. Quality of life is much more important to these individuals than quantity (Nash et al., 2021; Sunner et al., 2021; Weeks et al., 2021).

This study has a number of limitations. The COVID-19 crisis is now in its third year and is still ongoing, although much more manageable. However, our data were primarily collected during the first year, so do not reflect how crisis management and related tasks developed since the first quarter of 2021. Nevertheless, we believe that our differentiation between the two phases of the crisis response remains relevant. Another limitation is that we could have included more respondents (seven) and collected data for longer, which would have strengthened our findings. Still, we conducted 47 interviews with our respondents plus a focus group meeting. Although we only interviewed nursing home directors and one dedicated crisis manager, we believe that our findings are broadly relevant to crisis management. The phases we identified in crisis response, the shift in crisis management tasks, and the additional types of work may be relevant to other long-shadow crises and to crisis management in other settings. However, future research will be required to substantiate this.

In conclusion, our study has shown that nursing home managers performed a number of tasks to deal with the COVID-19 crisis, namely, diagnosing and deciding, mobilizing and organizing, containing and mitigating, informing and empowering, and coordinating and collaborating. We found that the response to this type of crisis can be distinguished into an acute phase and an adaptive phase. The acute phase is characterized by saving lives, centralized decision-making, compliancy, and solidarity. In contrast, the adaptive phase is characterized by reflection, decentralized decision-making, and competing values and perspectives. As a result, we identified a change of “color” when going from one phase to the other. We also identified three types of work that are relevant to this type of crisis: resilience work, emotion work, and normative work. Making sense of the crisis management tasks in this type of crisis, observing how these tasks emerge, and distinguishing the three types of work involved has helped us to understand the dynamics of crisis management.
